# Incidence of post-obturation pain after single-visit versus multiple-visit non-surgical endodontic treatments

**DOI:** 10.1186/s12903-015-0082-y

**Published:** 2015-08-14

**Authors:** Amy Wai-Yee Wong, Shinan Zhang, Samantha Kar-Yan Li, Xiaofei Zhu, Chengfei Zhang, Chun-Hung Chu

**Affiliations:** 1 Faculty of Dentistry, The University of Hong Kong, Hong Kong, China; 2 VIP Dental Service & Geriatric Dentistry, School and Hospital of Stomatology, Peking University, Beijing, China

## Abstract

**Background:**

Post-obturation pain is frustrating to both patients and dentists. Its incidence may change with the use of contemporary endodontic techniques. This randomised clinical trial aims to compare the incidence of post-obturation pain at one and seven days after single-visit and multiple-visit non-surgical endodontic treatments.

**Methods:**

Patients who required primary endodontic treatment in the two clinical trial centres in Hong Kong (HK) and in Beijing (PK) were recruited. Three HK dentists and three PK dentists performed endodontic treatments on 567 teeth using the same procedures and materials, either in a single visit or over multiple visits, using either core carrier or cold lateral condensation for obturation.

**Results:**

The attrition rate was 5.1 %, and a total of 538 teeth were evaluated. Among these teeth, 232 (43 %) were operated in HK, 275 (51 %) were treated in a single visit, and 234 (43 %) were treated using core carrier obturation. Logistic regression analysis showed that teeth with apical periodontitis (OR = 0.35, 95 % CI = 0.21–0.57, *p* < 0.01) and less pre-operative pain (OR = 1.10, 95 % CI = 1.03–1.18, *p* < 0.01) had lower incidences of post-obturation pain after one day. The incidences of post-obturation pain after one day for single-visit and multiple-visit treatments were 24.7 % (68 of 275) and 33.5 % (88 of 263), respectively (*p* = 0.50). The incidences of post-obturation pain after seven days for single-visit and multiple-visit treatments were 4.0 % (11 of 275) and 5.3 % (14 of 263), respectively (*p* = 0.47).

**Conclusions:**

There was no significant difference in the incidences of post-obturation pain after one day and seven days with single-visit or multiple-visit endodontic treatments.

**Trial registration:**

ChiCTR-IOR-15005989

## Background

Patients commonly complain of post-obturation discomfort and pain after endodontic (root canal) treatments, which can upset both clinicians and patients. The pain intensity can range from mild to severe, and it is widely described as occurring in flare-ups. The duration of the pain can range from one day to several weeks and can be a major cause of patient dissatisfaction. In addition, post-obturation pain after endodontic treatment is a poor indicator of pathosis and an even more unreliable predictor of long-term success [1]. The reported findings on post-obturation pain differ between studies. A systematic review found that it occurs in around 4 to 10 % of patients, in general [2]. However, DiRenzo and colleagues reported in their review that the incidence of post-obturation pain after non-surgical endodontic treatment can be greater than 50 % [1].

Endodontic treatment once necessitated multiple visits, as it required a considerable amount of time to complete [2]. Multiple-visit root canal treatment is accepted as a safe and common therapy. However, the rationale for multiple-visit endodontic treatment is being questioned. A systematic review found no significant differences between the antimicrobial efficacies reported for single-visit and multiple-visit treatments [2]. In addition, the use of contemporary endodontic techniques and equipment, such as magnifying devices, electronic apex locators, and engine-driven rotary nickel titanium files, not only increases the success rate of endodontic treatment but also shortens the time needed for treatment [3].

Cold lateral condensation (CLC) using gutta-percha is a commonly taught method of obturation. Dental practitioners use it often, and it frequently serves as a basis of comparison for new obturation techniques [4]. The core carrier obturation technique has become popular since its introduction in late 1980s, as studies have generally found that it as effective as CLC for root canal obturation [4–6]. In addition, many clinicians consider it to be fast, predictable, easy to use, effective, and useful for small, curved, or densely packed canals [7]. The Thermafil (TF) obturator (Dentsply Maillefer, Ballaigues, Switzerland) is a typical product used in core carrier obturation [4]. Gencoglu compared the apical sealing of obturation with TF and CLC and found that TF was better than CLC [8]. Studies have also suggested that core carrier obturation is more effective than CLC at filling lateral canals [9, 10]. Furthermore, TF was shown to have less leakage than CLC [8, 11].

If the incidence and intensity of post-obturation pain and the long-term success rate for single-visit and multiple-visit endodontic treatments are similar, single-visit treatment can be considered to be the more comfortable and efficient option. The aim of this study was to compare the incidence of post-obturation pain at one and seven days after single-visit and multiple-visit primary non-surgical endodontic treatments. The primary outcome measured was the incidence of post-obturation pain. The secondary outcome measured was the intensity of post-obturation pain. The first hypothesis is that there is no difference in the incidence of post-obturation pain for single-visit and multiple-visit non-surgical endodontic therapies one day after obturation; the second hypothesis is that there is no difference in the incidence of post-obturation pain for single-visit and multiple-visit non-surgical endodontic therapies seven days after obturation.

## Methods

### Patient recruitment

The study was approved by the Institutional Review Board of the University of Hong Kong/Hospital Authority Hong Kong West Cluster (HKU UW 09 - 303) in Hong Kong and the Institutional Review Board of Peking University (PKU IRB 00001052 - 10071) in Beijing, China. The study is registered at the World Health Organization’s International Clinical Trials Registry Platform (Clinical trial registration no.: ChiCTR-IOR-15005989). Chinese patients aged 18 or above who were generally healthy and required primary endodontic treatment via the University of Hong Kong (HKU) Health Service Dental Clinic or the Peking University (PKU) School and Hospital of Stomatology Special Service Clinic in Beijing were invited to participate in the study. Teeth with pulpotomy were not accepted, and at least half of the coronal structure had to be present. The protocol of the study was explained to the participants, and consent was obtained. Patients who had severe acute pulpitis, facial swelling or systemic infection, severe systemic disease, increased stress on the temporomandibular joint musculature, or increased psychological stress were excluded from this study.

**Fig. 1:**
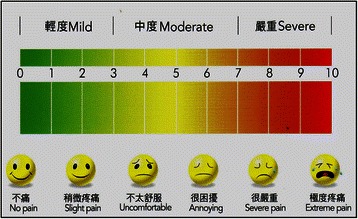
The chart used for the patient’s evaluation of post-obturation pain

**Fig. 2:**
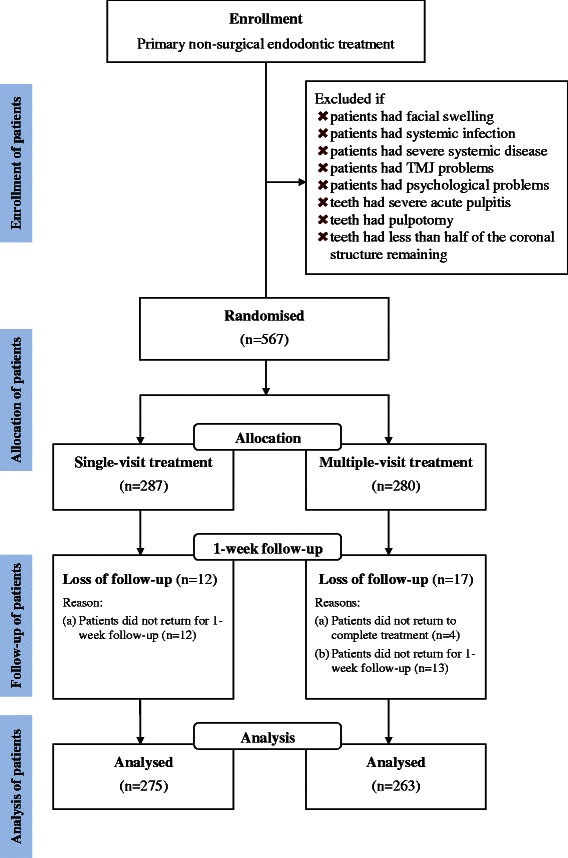
The study’s flow chart

Participants were scheduled for endodontic treatment. The preoperative clinical signs were recorded, including the presence of apical periodontitis (via the presence of apical radiolucency in the radiograph), chronic apical abscesses with or without the sinus tract, tooth mobility (MII, i.e., 1 mm horizontal mobility or above), tenderness to percussion, pockets, and preoperative pain. The pain assessment was adapted from our previous study [12], which measured pain on a 10-point Likert scale, ranging from no pain (score 0) to extreme pain (score 10), as shown in Fig. 1. Patients were reviewed one week after the obturation of the root canals, during which the presence of the clinical signs mentioned above were assessed and recorded. They were also asked about their 1-day and 7-day post-obturation pain, using assessment scale mentioned above (Fig. 1). Figure 2 is the study’s flow chart.

### Sample size calculation

The primary outcome measured was the prevalence of post-obturation pain, which was used to calculate the sample size. The prevalence of post-obturation pain was estimated at 10 %. We considered a difference of at least 10 % between single-visit and multiple-visit endodontic treatments to be clinically significant and statistically achievable. The estimated sample size was based on the expected prevalence of post-obturation pain, with the power of the study set at 80 % (β = 0.20) and with α = 0.05 as the significance level. Using the software program G*Power, version 3.1.7 (Franz Faul, Kiel University, Germany), we calculated that at least 199 teeth would be required per study group. We estimated the response rate to be 85 % and therefore aimed to recruit at least 230 teeth per group in this study.

### Clinical procedure

Three dentists from HKU and three dentists from PKU formed three pairs of dentists with similar clinical experience, to carry out the endodontic treatments. One dentist from each pair was trained to use the magnifying loupe (2.5×). All six dentists performed standardised endodontic treatments, which were done over either a single visit or multiple visits, and the obturations were performed with either CLC or TF. Receptionists randomly assigned patients to the dentists for treatment using the random-number generating function of a calculator. The dentists received a training workshop prior to this clinical trial to standardise their instrumentation and obturation technique, as described below. Local anaesthetic was given, and a rubber dam was used for isolation. The access cavity was prepared with a glide path before the use of rotary instruments. The root canals were prepared using a crown-down technique, which prepares the coronal part of the canal before the apical portion to achieve a straight-lined access for rotary nickel-titanium endodontic files (ProTaper Universal, Dentsply Maillefer, Ballaigues, Switzerland). Sodium hypochlorite solution at 5.25 % combined with a chelating agent of 17 % EDTA solution was used for irrigation. EDTA 15 % lubricant (RC-Prep, Premier, Philadelphia, USA) was used in the shaping procedures [13]. The working length was measured by an electronic apex locator (Root ZX, J Morita, Kyoto, Japan). The apical third of the canals was instrumented with F2 file with a diameter #25 at its tip. The canal was obturated after preparation if the tooth was assigned to the single-visit group. Otherwise (for the multiple-visit group), non-setting 35 % calcium hydroxide paste (UltraCal XS, Ultradent, South Jordan, UT, USA) was used as inter-appointment medication. The tooth was temporarily restored with resin-modified zinc oxide and eugenol cement (IRM, LD Caulk Dentsply, Milford, CT, USA) until obturation. The prepared tooth was obturated on the subsequent visit, which was usually seven days later. The root-canal sealer (AH Plus, Dentsply DeTrev GmbH, Konstanz Germany) was applied prior to obturation with either TF or CLC. The obturated teeth were then sealed with resin-modified zinc oxide and eugenol cement on the same visit. All the treated teeth, including single-rooted and multi-rooted teeth, received the same procedures.

### Data analysis

The collected data were entered into a personal computer and analysed with the IBM SPSS Statistics 21.0 program (Armonk, NY, USA). The primary outcome of this research was to evaluate the prevalence of pain at one day and seven days after obturation among the single-visit and multiple-visit treatment groups. The following possibly related independent variables were also considered: patients’ age and gender (male or female), tooth vitality (vital or non-vital), number of canals (single or multiple), tooth position (anterior or posterior), arch (upper or lower), obturation method (CLC or TF), use of magnifying loupe (yes or no), status of opposing tooth (yes or no), abscess or sinus tract (yes or no), tender to percussion (yes or no), tooth hypermobility (yes or no), periodontal pocket (yes or no), apical periodontitis (yes or no), C-shaped canal (yes or no), operator’s experience (≤10 years or >10 years), intensity of pre-operative pain (0 to 10), and treatment time. For the categorical independent variables, separated chi-square tests were used to assess the differences in the prevalence of pain among the groups. For the continuous independent variables, separated two-sample t-tests were used to assess the mean difference between the groups with and without post-obturation pain. The significant factors in the above tests (*p* < 0.05) were assessed using multiple logistic regression to investigate their relationships with post-obturation pain after one day and after seven days.

Separated analysis of variance (ANOVA) regression models were used to study the relationships between the intensity of post-obturation pain among the treatment group and the previously mentioned independent variables. The significant variables (*p* < 0.05) were then entered into the ANCOVA model for analysis. The level of statistical significance was set at 5 %, and all of the statistical analyses involved 2-tailed tests.

## Results

A total of 538 out of 567 teeth were evaluated, with 275 teeth (51 %) receiving single-visit endodontic treatment. Table 1 shows the descriptive statistics for the independent variables according to treatment group. The mean (±SD) age of the patients was 46.5 ± 17.0, and 305 (57 %) were female. Among the 538 teeth, 219 (41 %) were vital, 219 (41 %) had a single root canal, 135 (25 %) were anterior (incisors or canines), 25 (5 %) had no opposing teeth, and 320 (59 %) were in the upper arch. There were 210 teeth (39 %) with apical periodontitis, 182 (34 %) that were tender to percussion, 57 (11 %) with a periodontal pocket at least 4 mm in depth, 50 (9 %) with a sinus tract or abscess, 22 (4 %) with a C-shaped canal, and 18 (3 %) with at least MII mobility. There were 306 teeth (57 %) done in PKU, 234 (43 %) obturated using TF, 243 (45 %) treated using loupes, and 309 (57 %) treated by dentists with less than or equal to 10 years of experience.

**Table 1:** Independent variables according to treatment groups (*Significant result, p < 0.05)

Independent variable	Category	Single-visit	Multiple-visit	*p*-value
		(*n* = 275)	(*n* = 263)	
		No. (Col %)	No. (Col %)	
Obturation method	Thermafil	155 (56 %)	79 (30 %)	<0.01*
	Lateral condensation	120 (44 %)	184 (70 %)	
Use of loupe	Yes	104 (38 %)	139 (53 %)	<0.01*
	No	171 (62 %)	124 (47 %)	
Operator’s experience	<=10 years	121 (44 %)	188 (71 %)	<0.01*
	>10 years	154 (56 %)	75 (29 %)	
Gender	Male	131 (48 %)	102 (39 %)	0.04*
	Female	144 (52 %)	161 (61 %)	
Arch	Upper	172 (63 %)	148 (56 %)	0.14
	Lower	103 (37 %)	115 (44 %)	
Tooth position	Anterior	92 (33 %)	43 (16 %)	<0.01*
	Posterior	183 (67 %)	220 (84 %)	
Canal	Single	142 (52 %)	77 (29 %)	<0.01*
	Multiple	133 (48 %)	186 (71 %)	
C-shaped canal	Yes	5 (2 %)	17 (6 %)	0.01*
	No	270 (98 %)	246 (94 %)	
Periodontal pocket	Yes	25 (9 %)	32 (12 %)	0.25
	No	250 (91 %)	231 (88 %)	
Apical periodontitis	Yes	110 (40 %)	100 (38 %)	0.64
	No	165 (60 %)	163 (62 %)	
Tender to percussion	Yes	74 (27 %)	108 (41 %)	<0.01*
	No	201 (73 %)	155 (59 %)	
Hypermobility	Yes	6 (2 %)	12 (5 %)	0.12
	No	269 (98 %)	251 (95 %)	
Abscess or sinus tract	Yes	23 (8 %)	27 (10 %)	0.45
	No	252 (92 %)	236 (90 %)	
Tooth vitality	Vital	101 (37 %)	118 (45 %)	0.05
	Non-vital	174 (63 %)	145 (55 %)	
Opposing tooth	Yes	261 (95 %)	252 (96 %)	0.62
	No	14 (5 %)	11 (4 %)	
Post-operative pain after 1 day	Pain	68 (25 %)	88 (33 %)	0.03*
	No pain	207 (75 %)	175 (67 %)	
Post-operative pain after 7 days	Pain	11 (4 %)	14 (5 %)	0.47
	No pain	264 (96 %)	249 (95 %)	
Independent variable	All cases	Single-visit	Multiple-visit	*p*-value
	Mean (SD)	Mean (SD)	Mean (SD)	
Treatment Time (*n* = 538)	51.07 (30.59)	44.85 (26.57)	57.57 (33.11)	<0.01*
Pre-operative pain intensity (*n* = 538)	2.04 (2.93)	1.18 (2.32)	2.93 (3.23)	<0.01*
Pain intensity after 1 Day (*n* = 156)	3.26 (1.72)	2.81 (1.54)	3.61 (1.78)	<0.01*
Pain intensity after 7 Days (*n* = 25)	2.60 (1.92)	1.64 (0.67)	3.36 (2.24)	0.02*
Age (*n* = 538)	46.5 (16.9)	46.69 (17.34)	46.29 (16.52)	0.78

Post-operative pain was found in 156 teeth (29 %) after one day. Most of the pain reported was mild to moderate both after one day (*n* = 149/156, 96 %) and after seven days (*n* = 23/25, 92 %). Table 2 shows the incidence of post-obturation pain after one day and seven days along with other variables. Teeth that were treated in a single visit were obturated with TF, were treated by an operator with >10 years’ experience, had apical periodontitis, were non-vital, and had lower levels of pre-operative pain all showed lower incidences of post-obturation pain after one day. Only 25 teeth (5 %) had post-obturation pain after seven days, and 21 of them were upper teeth. The post-obturation pain after seven days was not related to the number of treatment visits (chi-square test, *p* = 0.47).

**Table 2:** Independent variables and post-obturation pain incidence after 1 day and 7 days (*n* = 538)

Independent variable	Category (No.)	Incidence of pain after 1 day			Incidence of pain after 7 days		
		No.	%	*p*-value	No.	%	*p*-value
Treatment visit	Single (275)	68	25 %	0.03*	11	4 %	0.47
	Multiple (263)	88	33 %		14	5 %	
Obturation method	Thermafil (234)	47	20 %	<0.01*	10	4 %	0.72
	Lateral condensation (304)	109	36 %		15	5 %	
Use of loupe	Yes (243)	74	30 %	0.50	10	4 %	0.59
	No (295)	82	28 %		15	5 %	
Operator’s experience	<=10 years (309)	110	36 %	<0.01*	15	5 %	0.79
	>10 years (229)	46	20 %		10	4 %	
Gender	Male (233)	65	28 %	0.62	10	4 %	0.73
	Female (305)	91	30 %		15	5 %	
Arch	Upper (320)	92	29 %	0.38	21	7 %	0.01*
	Lower (218)	64	29 %		4	2 %	
Tooth position	Anterior (135)	34	25 %	0.26	5	4 %	0.55
	Posterior (403)	122	30 %		20	5 %	
Canal	Single (219)	59	27 %	0.38	10	5 %	0.94
	Multiple (319)	97	30 %		15	5 %	
C-shaped canal	Yes (22)	8	35 %	0.44	0	6 %	0.62^a^
	No (516)	148	24 %		25	3 %	
Periodontal pocket	Yes (57)	17	30 %	0.88	5	9 %	0.17^a^
	No (481)	139	29 %		20	4 %	
Apical periodontitis	Yes (210)	36	17 %	<0.01*	7	3 %	0.25
	No (328)	120	37 %		18	5 %	
Tender to percussion	Yes (182)	60	33 %	0.15	13	7 %	0.05^a^
	No (356)	96	27 %		12	3 %	
Hypermobility	Yes (18)	5	28 %	0.91	3	17 %	0.05
	No (520)	151	29 %		22	4 %	
Abscess or sinus tract	Yes (50)	10	20 %	0.14	4	8 %	0.28^a^
	No (488)	146	30 %		21	4 %	
Tooth vitality	Vital (219)	78	36 %	0.01*	12	5 %	0.45
	Non-Vital (319)	78	24 %		13	4 %	
Opposing tooth	Yes (513)	150	29 %	0.57	24	5 %	1.00^a^
	No (25)	6	24 %		1	4 %	
Independent variable		Incidence of pain after 1 day			Incidence of pain after 7 days		
		No (*n* = 382)	Yes (*n* = 156)	*p*-value	No (*n* = 513)	Yes (*n* = 25)	*p*-value
		Mean (SD)	Mean (SD)		Mean (SD)	Mean (SD)	
Age		46.9 (17.2)	45.4 (16.1)	0.36	46.5 (16.9)	46.4 (17.9)	0.97
Pre-operative pain intensity		1.7 (2.7)	2.8 (3.2)	<0.01*	2.0 (2.9)	2.4 (2.8)	0.57
Treatment time		52.5 (31.3)	47.6 (28.4)	0.09	50.7 (29.9)	59.1 (42.9)	0.34

^a^Fisher’s exact test; *Significant result, p < 0.05

Multiple logistic regression (full model) showed that the number of treatment visits was not related to the incidence of post-obturation pain after one day when adjusting for other possible related variables (adjusted *p* = 0.50) (Table 3). The incidence of post-obturation pain after one day was lower for teeth with the presence of apical periodontitis (OR = 0.35, 95 % CI = 0.21–0.57, *p* < 0.01). In addition, teeth with more intense pre-operative pain had increased incidence of post-obturation pain after one day (OR = 1.10, 95 % CI = 1.03–1.18, *p* < 0.01), with Nagelkerke R-squared = 0.11.

**Table 3:** Multiple logistic regression on post-obturation pain incidence after 1 day (*n* = 538)

Independent variable	Category	Odds ratio	95 % C.I.	*p*-value
Treatment visit	Single	0.87	0.57 - 1.32	0.50
	Multiple	1		
Obturation method	Thermafil	0.38	0.04 - 3.85	0.41
	Lateralcondensation	1		
Operator’s experience	<=10 years	0.68	0.07 - 7.12	0.75
	>10 years	1		
Apical periodontitis	Yes	0.35	0.21 - 0.57	<0.01*
	No	1		
Vitality	Vital	0.67	0.40 - 1.11	0.12
	Non-Vital	1		
Pre-operative pain intensity	0–10	1.10	1.03 - 1.18	<0.01*

Nagelkerke R-squared = 0.11;  *Significant result, p < 0.05

Table 2 shows that the arch was the only significant variable associated with the incidence of post-obturation pain after seven days. Further simple logistic regression found that endodontically treated upper teeth had increased incidence of post-obturation pain after seven days (OR = 3.76, 95 % CI = 1.27–11.10, *p* = 0.02), with Nagelkerke R-squared = 0.04.

Table 4 shows the factors related to pain intensity after one day in the separated unadjusted models and the adjusted model (a multi-way ANCOVA model). The mean (±SD) pain intensity was 3.26 ± 1.72 among the 156 endodontically treated teeth with post-obturation pain after one day. The post-obturation pain intensity was related to the treatment group, presence of an abscess or sinus tract, percussion tenderness, use of a magnifying loupe, obturation method, and intensity of the pre-obturation pain (Table 4). Multiple-way ANCOVA analysis found that multiple-visit endodontic treatment, presence of an abscess or sinus tract, obturation with TF, and more severe pre-operative pain showed increased intensity of post-obturation pain after one day.

**Table 4:** Multi-way ANCOVA on post-obturation pain intensity after 1 day (*n* = 156) (*Significant result, p < 0.05)

Independent variable	Unadjusted model			Adjusted model		
	Estimate	95 % C.I.	*p*-value	Estimate	95 % C.I.	*p*-value
Single-visit	-0.80	-1.34 - -0.27	<0.01*	-0.79	-1.34 - -0.23	0.01*
Thermafil (Obturation method)	0.78	0.20 - 1.36	0.01*	0.96	0.32 - 1.60	<0.01*
Use of loupe	0.68	0.15 - 1.22	0.01*	0.33	-0.23 - 0.90	0.24
Operator’s experience < =10 years	-0.58	-1.17 - 0.01	0.05			
Male (Gender)	0.10	-0.45 - 0.66	0.71			
Upper teeth	0.07	-0.48 - 0.63	0.79			
Anterior teeth	-0.19	-0.85 - 0.47	0.58			
Single canal	0.34	-0.22 - 0.9	0.23			
C-shaped canal	-1.20	-2.42 - 0.02	0.05			
Periodontal pocket	0.30	-0.58 - 1.17	0.50			
Apical periodontitis	0.34	-0.30 - 0.99	0.29			
Tender to percussion	0.66	0.11 - 1.21	0.02*	-0.5	-1.19 - 0.19	0.15
Hypermobility	0.35	-1.20 - 1.90	0.66			
Abscess or sinus tract	1.75	0.67 - 2.83	<0.01*	1.49	0.42 - 2.56	0.01*
Tooth vitality	-0.50	-1.04 - 0.04	0.07			
Opposing tooth	1.31	-0.09 - 2.72	0.07			
Age	0.00	-0.01 - 0.02	0.68			
Pre-operative pain intensity	0.12	0.04 - 0.20	<0.01*	0.11	0.02 - 0.20	0.02*
Treatment time	0.01	0.00 - 0.02	0.11			

Table 5 shows the factors related to pain intensity after seven days in separated, unadjusted models (ANOVA regression models). Among the 25 endodontically treated teeth with post-obturation pain after seven days, the mean score (±SD) of the pain intensity was 2.60 ± 1.92. Teeth that received single-visit endodontic treatments were less painful after seven days (*p* = 0.02).

**Table 5:** Post-obturation pain intensity after 7 days (*n* = 25)

Independent variable	Estimate	95 % C.I.	*p*-value
Single-visit	-1.72	-3.17 - -0.27	0.02 *
Thermafil (Obturation method)	-0.17	-1.82 - 1.48	0.84
Use of loupe	0.33	-1.31 - 1.98	0.68
Operator’s experience < =10 years	0.17	-1.48 - 1.82	0.84
Male (Gender)	0.00	-1.65 - 1.65	1.00
Upper teeth	0.12	-2.09 - 2.33	0.91
Anterior teeth	-0.25	-2.27 - 1.77	0.80
Single canal	0.50	-1.14 - 2.14	0.53
C-shaped canal	NA^a^		
Periodontal pocket	-1.25	-3.20 - 0.70	0.20
Apical periodontitis	1.15	-0.58 - 2.88	0.18
Tender to percussion	-0.13	-1.75 - 1.49	0.87
Hypermobility	0.08	-2.41 - 2.57	0.95
Abscess or sinus tract	1.96	-0.07 - 4.00	0.06
Tooth vitality	-1.15	-2.70 - 0.39	0.14
Opposing tooth	1.67	-2.40 - 5.73	0.41
Age	-0.04	-0.08 - 0.00	0.06
Pre-operative pain intensity	-0.08	-0.38 - 0.21	0.56
Treatment time	0.00	-0.02 - 0.02	0.80

^a^Not applicable; no C-shaped canal found on the 25 patients; *Significant result, p < 0.05

## Discussion

Endodontic treatment is reasonable, based on the treatment cost; operators and patients consider it to be a practical clinical technique to resume the function of treated teeth [14]. Most patients are concerned about the pain encountered during and after endodontic treatment. Both patients and operators are keen to identify the factors that increase the probability of post-obturation pain. In treatment planning, it is helpful to be aware of the risks associated with post-treatment pain. Operators can prepare effectively via communication with patients before treatment and can also apply a different approach to deal with patients who experience post-obturation pain [15].

The null hypothesis that there is no difference in the incidence of post-obturation pain of single-visit versus multiple-visit non-surgical endodontic therapies at one day and seven days after obturation is supported by the results of this randomised clinical trial. This lack of difference suggests that single-visit treatment is an acceptable alternative to the conventional multiple-visit treatment when post-obturation discomfort is the concern. Regarding the success rate, a recent systematic review concluded that the success rates of single-visit and multiple-visit non-surgical endodontic therapies are similar [2]. If the patient can endure a longer treatment procedure, then single-visit endodontic treatment is generally considered to be more comfortable and efficient than multiple-visit treatment.

Post-obturation pain was assessed after one day and seven days in this study. Several studies reported pain after two hours [16], four hours [17] and seven days [18, 19]. The reported incidence of pain ranged from 4 [20] to 87 % [21]. Studies have shown that the pain intensity was highest on the first day and dropped afterwards [20]. Another study found that post-obturation pain could persist after seven days but that its intensity typically went down significantly [22]. Therefore, this study evaluated post-obturation pain after one day and seven days. The results showed that the incidence of post-obturation pain was fairly common after one day (29 %) but that it mostly subsided after seven days.

There was no significant difference in the incidence of post-obturation pain between single-visit and multiple-visit treatments, which is in agreement with previous studies [1, 18, 19, 23–33]. Furthermore, this study found that post-obturation pain after one day and seven days was more severe for teeth that received multiple-visit treatment than for those receiving single-visit treatment. This finding concurs with previous studies reporting that short-term post-obturation pain was significantly higher in patients receiving multiple-visit endodontic treatment than in patients receiving single-visit treatment [34, 35].

In this study, calcium hydroxide – the most commonly used intra-canal medicament – was used. Studies have reported no significant difference in post-obturation pain with the use of calcium hydroxide compared to the use of other intra-canal medicaments [36, 37]. Sodium hypochlorite solution was used for irrigation. Bashetty and Hegde reported that the type of irrigant used had no association with the post-obturation pain after one day or after seven days [38]. Another study found no relationships between the incidence of post-obturation pain and the two working-length determination methods – via electronic apex locator and digital radiography [39]. Rotary instruments with nickel-titanium files were used in this clinical trial. Several studies reported that the incidence of post-obturation pain after rotary canal preparation was less than after preparation with manual instrumentation [40–42]. However, post-obturation pain, if any, could last longer with rotary preparation than with hand instrumentation [40]. Aqrabawi and Jamani did not find significant differences in post-obturation pain using stainless steel versus nickel-titanium endodontic files [43]. Silva and colleagues found that foraminal enlargement would not significantly affect post-obturation pain [44].

The purpose of this study was to compare the incidence of post-obturation pain for single-visit and multiple-visit primary non-surgical endodontic treatments. A number of confounding factors were recorded. Logistic regression was performed to explore their association with the incidence and intensity of post-obturation pain. These factors include gender, age, the operator’s experience, obturation method, use of loupe, arch (upper or lower), tooth position (anterior or posterior), presence of an opposing tooth, C-shaped canal, and tooth status (which includes tooth vitality, the presence of a periodontal pocket, apical periodontitis, percussion tenderness, hypermobility, and abscess or sinus tract). There was no significant difference in post-obturation pain based on age or gender in this study, which is supported by other clinical studies [35, 45]. Some potential confounding factors, such as the quality of obturation and canal adaptation, were not recorded or analysed in this study. Due to this limitation, this study’s results should be interpreted with care.

In this study, obturation with TF reduced the incidence of post-obturation pain after one day. It was plausible that significantly less obturation force was used in Thermafil obturation than in cold lateral condensation [46, 47]. However, the pain associated with Thermafil obturation may have higher intensity than that of cold lateral condensation. The results agreed with a previous study finding that Thermafil resulted in significantly higher levels of pain than cold lateral condensation [16]. Albashaireh and Alnegrish reported that endodontic treatment on non-vital teeth had a higher chance of developing post-obturation pain than on vital teeth [33]; however, Gotler and colleagues reported the opposite, with more post-obturation pain in vital teeth [48]. We could not find a significant association between post-obturation pain and the tooth’s vitality status. These findings agreed with several previous studies [20, 24, 26, 27, 35, 49]. This study also found that there was no significant association between post-obturation pain and the number of roots, which was in agreement with the studies by Raju *et al.* and Wang *et al.* [18, 24].

This study found no relationship between post-obturation pain and the condition of opposing teeth, which agreed with a previous study [50]. Several studies reported that mandibular teeth had a higher chance of post-obturation pain [51, 52]; however, we found more post-obturation pain in maxillary teeth after seven days. We found more severe post-obturation pain after one day in teeth with no apical periodontitis, which was in agreement with a previous study [29]. The phenomenon might be explained by a lack of available periapical space for resolution after inflammation [29]. This study found that teeth with pre-operative pain increased the risk of post-obturation pain, which was in agreement with previous studies [20, 29, 35, 45, 51, 53–57].

There was no significant difference in post-obturation pain or pain intensity due to the operator’s experience. It was interesting to find contradicting results regarding the effects of the operator’s experience on post-obturation pain. One study reported significantly lower post-obturation pain among patients of undergraduate operators compared with those of residents or faculty members due to the extended time spent working on disinfecting canals during instrumentation [58]. Another study reported no significant difference in post-obturation pain due to the operator’s experience. The differences found in the above studies did not take into consideration the cases’ relative difficulty.

It is generally agreed that the operator’s clinical experience will affect the success rate and post-obturation pain. However, this study found no association between operator’s experience and post-obturation pain or pain intensity. Law and colleagues found that the effect of clinical experience on post-obturation pain was difficult to determine [57]. The choice between single-visit and multiple-visit treatments was based on the operator’s skill. Some clinicians have suggested multiple-visit treatment when in doubt. For communities where patients tended to fail to attend subsequent appointments once the pain was relieved on the first appointment, single-visit treatment is regarded as a safe and effective alternative to incomplete multiple-visit treatment [59].

## Conclusions

In this randomised clinical trial, post-obturation pain after non-surgical endodontic therapy was not uncommon after one day (29 %), but only 5 % of teeth had pain after seven days. There was no significant difference in the incidences of post-obturation pain after one day and seven days among single-visit and multiple-visit endodontic treatments. Among the teeth with post-obturation pain, the single-visit group had lower-intensity pain, after one day and after seven days, than the multiple-visit group had.
